# New Mitochondrial Gene Rearrangement in *Psyttalia concolor*, *P. humilis* and *P. lounsburyi* (Hymenoptera: Braconidae), Three Parasitoid Species of Economic Interest

**DOI:** 10.3390/insects11120854

**Published:** 2020-12-02

**Authors:** Chanté Powell, Virgilio Caleca, Clint Rhode, Luis Teixeira da Costa, Barbara van Asch

**Affiliations:** 1 Department of Genetics, Stellenbosch University, Private Bag X1, Matieland 7602, South Africa; chantepowell4@gmail.com (C.P.); clintr@sun.ac.za (C.R.); 2 Department of Agricultural, Food and Forest Sciences, Università degli Studi di Palermo, Viale delle Scienze, Edificio 5, 90128 Palermo, Italy; virgilio.caleca@unipa.it; 3 Unit for Cardiac and Cardiovascular Genetics, Department of Medical Genetics, Oslo University Hospital, Postboks 4956 Nydalen, 0424 Oslo, Norway; 4 Norsk Entomologisk Forening, Naturhistorisk Museum, Universitetet i Oslo, Postboks 1172 Blindern, 0318 Oslo, Norway

**Keywords:** braconidae, cyclostome wasps, mitogenomics, opiinae, olive, phylogeny, *Psyttalia humilis*, *Psyttalia lounsburyi*

## Abstract

**Simple Summary:**

Parasitoid wasps in the family Braconidae are generally highly specialized and can be used as agents for biological control of arthropod pests. *Psyttalia concolor, Psyttalia humilis* and *Psyttalia lounsburyi* parasitize the larvae of the olive fruit fly (*Bactrocera oleae*), the most damaging pest of cultivated olives in the world. *Psyttalia concolor* is native to the Mediterranean, and *P. humilis* and *P. lounsburyi* are native to sub-Saharan Africa. Despite their potential for agricultural pest control, these species have been poorly characterized at the genetic level. We sequenced the mitochondrial genome of the three species and compared its organization with other Braconidae. *Psyttalia* had a unique gene rearrangement involving the positions of transfer RNA genes. We also present a phylogenetic reconstruction of the Braconidae and confirm the phylogenetic placement of *Psyttalia* in the subfamily Opiinae.

**Abstract:**

The family Braconidae consists mostly of specialized parasitoids, some of which hold potential in biocontrol of agricultural pests. *Psyttalia concolor, Psyttalia humilis* and *Psyttalia lounsburyi* are parasitoids associated with *Bactrocera oleae*, a major pest of cultivated olives. The native range of *Psyttalia concolor* is the Mediterranean, and *P. humilis* and *P. lounsburyi* are native to sub-Saharan Africa. This study reports the mitochondrial genomes of the three species, thus laying the foundation for mitogenomic analyses in the genus *Psyttalia*. Comparative mitogenomics within Braconidae showed a novel gene arrangement in *Psyttalia* in involving translocation and inversion of transfer RNA genes. The placement of *Psyttalia* in the subfamily Opiinae was well-supported, and the divergence between *Psyttalia* and its closest relative (*Diachasmimorpha longicaudata*) was at ~55 MYA [95% highest posterior density (HPD): 34–83 MYA]. *Psyttalia lounsburyi* occupied the most basal position among the three *Psyttalia*, having diverged from the other two species ~11 MYA (95% HPD: 6–17 MYA). *Psyttalia concolor* and *P. humilis* were recovered as sister species diverged at ~2 MYA (95% HPD: 1.1–3.6 MYA). This phylogeny combining new sequences and a set of 31 other cyclostomes and non-cyclostomes highlights the importance of a comprehensive taxonomic coverage of Braconidae mitogenomes to overcome the lack of robustness in the placement of several subfamilies.

## 1. Introduction

The family Braconidae is a species-rich group that includes 40 subfamilies represented by over 1000 genera and more than 19,000 known species [1,2]. Braconidae are mostly composed of highly specialized parasitoids, and the majority of the subfamilies therein are ectoparasitic idiobionts (i.e., the host is unable to recover after the paralysis induced by the ovipositing wasp), or endoparasitic koinobionts (i.e., the host can recover after oviposition and develop normally, completing all larval instars) [3,4]. In general, Braconidae exhibit host-specific relationships at the subfamily level; however, this is less true for ectoparasitoids [1,4]. For example, the endoparasitic Microgastrinae attack only lepidopteran larvae, with the exception of one species associated with Trichoptera [5], and the endoparasitic Helconinae parasitize coleopteran larvae. In contrast, the ectoparasitic Braconinae attack a variety of holometabolous larvae, and the subfamily has been used as a model for studying the evolutionary transition between ecto- and endoparasitism [1,4]. Braconidae are divided into two major groupings of subfamilies: the cyclostomes and the non-cyclostomes. Cyclostomes are distinguished by a cavity above the mandible (hypoclypeal depression) which is a synapomorphy of the group, and comprise all the ectoparasitoids, some endoparasitoids and all known phytophagous braconids [1,3]. The cyclostome complex has been reported as monophyletic based on morphology [6,7], with the remaining non-cyclostome subfamilies as a sister clade based on integrated molecular and morphological data [8]. Molecular studies using the mitochondrial 16s rRNA and the nuclear 28s rRNA genes also recovered cyclostomes as monophyletic [9,10,11]. However, the phylogenetic relationships within cyclostomes have not been recovered with high statistical support, despite extensive taxon coverage [3]. Although the family Braconidae has received considerable taxonomic attention in recent years, substantial confusion persists over the definitions of several subfamilies, especially among cyclostomes [8].

The olive fruit fly *Bactrocera oleae* (Rossi, 1790) (Diptera: Tephritidae) has been a major pest of cultivated olives in the Mediterranean Basin since historical times. More recently, the species became an important threat to olive production in California where it quickly spread after the invasion was first detected in 1998 [12]. The olive fruit fly is controlled using primarily insecticides, which have limited success and negatively impact the environment [13]. Moreover, conventional pest control has been associated with increased frequency of insecticide resistance alleles in olive fruit fly populations [14,15,16,17]. Efforts to find agents for the biocontrol of *B. oleae* started over 100 years ago, and surveys for natural enemies have been conducted in South Africa, Namibia, Kenya, La Réunion, Canary Islands, Morocco, Pakistan, India and China [18]. The highest species diversity of parasitoid wasps (Braconidae and Chalcidoidea) associated with olive fruit flies in a single geographic region was found in the Western Cape province of South Africa, on native African wild olives [*Olea europaea* L. subsp. *cuspidata* (Wall ex G. Don Cif.)] [19,20]. The assemblage included four Braconidae koinobiont endoparasitoids endemic to sub-Saharan Africa: *Bracon celer* (Szépligeti, 1913), *Utetes africanus* (Szépligeti, 1910), *Psyttalia humilis* (Silvestri, 1913) and *Psyttalia lounsburyi* (Silvestri, 1913).

*Psyttalia lounsburyi* was described by Silvestri (1913) as a parasitoid of olive fruit flies on African wild olives in South Africa. *Psyttalia lounsburyi* was found to be genetically distinct from *P. humilis* (see below) and from their Mediterranean counterpart *Psyttalia concolor* (Szépligeti, 1910) [21]. *Psyttalia lounsburyi* has been reported in Kenya and South Africa, where it was recovered from *B. oleae* infesting wild olives [20,22]. Therefore, *P. lounsburyi* is thought to be a sub-Saharan Africa parasitoid specializing in *B. oleae*. However, the fact that it also accepts *Ceratitis capitata* (Wiedemann, 1824) as a host under laboratory conditions raises the possibility that it can parasitize other *Bactrocera* spp. in the wild, particularly *Bactrocera biguttula* (Bezzi, 1922), a close relative of *B. oleae* known to utilize African wild olives in South Africa, Namibia, and Kenya [21,23,24], and *B. munroi* White, 2004 also found it in Kenya on African wild olives [25]. *Psyttalia humilis* was described by Silvestri (1913) based on specimens reared from pears infested by *C. capitata* in Constantia, Cape Town (South Africa). The species has been reared from *B. oleae* collected from African wild olives in Kenya and South Africa [20,24,25]. *Psyttalia humilis* is morphologically indistinguishable from the Mediterranean *P. concolor* and has sometimes been treated as its junior synonym [26]. However, the fact that *P. humilis* has been recorded only in sub-Saharan Africa and *P. concolor* only in the in the Mediterranean Basin, and the genetic divergence found in DNA analyses across the genus *Psyttalia,* supports that *P. humilis* and *P. concolor* can be treated as separate species [21]. *Psyttalia concolor* is an endoparasitoid of *B. oleae* found on wild and cultivated olives in the Mediterranean region. *Psyttalia concolor* was first identified as an olive fruit fly parasitoid in Tunisia, and is also considered native to Sicily, southern Sardinia and southern Calabria [27,28]. More recently, the parasitoid was found in various areas of coastal Tuscany [29]. *Psyttalia concolor* has also reportedly been reared from medfly (*C. capitata*) infesting argan fruit (*Argania spinosa* L., Sapotaceae) in Morocco [30]. However, those specimens were not subjected to DNA-based analyses, and the indistinguishability between *P. humilis* and *P. concolor* demands caution in the identification of *Psyttalia*, especially if emerged from hosts other than *B. oleae*, which is presently the only confirmed host of *P. concolor* [21].

*Psyttalia concolor* has been used in trials for biological control of the olive fruit fly in the Mediterranean [31,32]. In 2003, California initiated a program focused on the evaluation and release of *P. humilis* and *P. lounsburyi*, but the introductions had limited success, as only *P. lounsburyi*, the most specialized of the two parasitoids was recovered [18,33,34].

Despite their potential utility and interesting evolutionary specialization as parasitoids of the olive fruit fly, *P. concolor*, *P. humilis* and *P. lounsburyi* have not been fully characterized at the level of the mitochondrial sequence. Insect mitochondrial genomes are powerful sources of information for the reconstruction of phylogenetic relationships due to their maternal inheritance, lack of recombination, conserved gene components and organization and relatively small size [35]. The genus *Psyttalia* (Walker, 1860) has not been represented in comparative mitogenomics, and its positioning within the family Braconidae has never been assessed in previous phylogenies using complete or near-complete mitogenome sequences [36,37,38]. Mitochondrial gene rearrangements can be particularly interesting in phylogenetic analyses because they occur frequently in certain groups of insects, including Hymenoptera, but are uncommon in closely related taxa [36,39,40]. Therefore, mitochondrial gene rearrangements provide additional information to help resolve deep phylogenetic nodes. A recent study explored the possibility of using mitogenome rearrangements to reconstruct phylogenetic relationships in Braconidae [37], and the results highlighted the importance of obtaining complete mitogenome sequences, as two regions previously known to harbor gene rearrangements in braconids [38] were not sequenced, thus potentially reducing the resolving power of the analysis. The present work lays the foundation for mitogenomics in the genus *Psyttalia*, and the clarification of the phylogenetic relationships of *P. concolor*, *P. lounsburyi* and *P. humilis* within Braconidae.

## 2. Materials and Methods

### 2.1. Sample Collection and Species Identification

**Figure 1:**
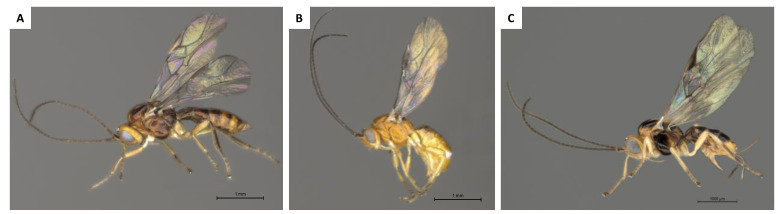
Adult specimens representative of three *Psyttalia* (Hymenoptera: Braconidae) species. (**A**) *Psyttalia concolor* male (scale bar = 1 mm); (**B**) *Psyttalia humilis* male (scale bar = 1 mm); and (**C**) *Psyttalia lounsburyi* female (scale bar = 100 μm).

Adult specimens of *P. humilis* and *P. lounsburyi* were reared from African wild olives (*Olea europaea* L. subsp. *cuspidata*) collected in April and May 2016 in Grahamstown (33.3195° S, 26.5171° E) and Stellenbosch (33.9951° S, 18.8676° E), respectively situated in the Eastern and the Western Cape province of South Africa. Adult specimens of *P. concolor* were reared from cultivated olives (*O. europaea* L. subsp. *europaea* var. *europaea*) collected in November 2014 in Constância (39.4781° N, 8.3372° W), in the Ribatejo province of Portugal. Morphological species identification was performed on ethanol-preserved adult specimens, using the taxonomic keys and photographic images available in the Parasitoids of Fruit-Infesting Tephritidae (PAROFFIT) database (http://paroffit.org), and previous descriptions [41] (Figure 1). Morphological species identification was confirmed by comparing DNA barcodes (650 bp; COI-5’) of *P. concolor*, *P. humilis* and *P. lounsburyi* and homologous sequences available on GenBank as of 18 November 2020. Intra- and interspecific genetic divergences were estimated as pairwise distances (p-distances) under the Kimura 2-parameter (K2P) model [42] in MEGA X [43]. Standard errors were calculated from 1000 bootstrap replicates.

### 2.2. DNA Extraction, Polymerase Chain Reaction (PCR) Amplification and Sequencing

Total DNA was extracted from a single adult specimen representative of each species using a standard SDS/Proteinase K method for *P. concolor,* and a standard phenol–chloroform method for *P. humilis* and *P. lounsburyi*.

#### 2.2.1. Sanger Sequencing

The complete mitogenome of *P. concolor* (15,308 bp) was obtained by Sanger sequencing, after PCR amplification in 18 overlapping segments, with shorter versions of four of these also used for sequencing (Table S1). All PCR and sequencing primers were designed by the authors specifically for this study except for segment S03*, which was amplified and sequenced using arthropod universal DNA barcoding primers [44]. PCR was carried out in 25 μL total volume containing 75 mM Tris-HCl (pH 8.8), 20 mM (NH_4_)_2_SO_4_, 0.01% (*v/v*) Tween 20 (Fermentas), 3.0 mM MgCl_2_ (Fermentas), 0.5 mM of each dNTP (Fermentas), 25 pmol of each primer (Macrogen) and 2.5 U of Taq DNA polymerase (Fermentas). Three hotstart and touchdown cycling protocols were used: (a) PAS1, consisting of 95 °C for 5 min; three cycles of 95 °C for 30 s; 60 °C for 1 min and 72 °C for 2 min; three cycles of 95 °C for 30 s; 57 °C for 1 min and 72 °C for 2 min; three cycles of 95 °C for 30 s; 54 °C for 1 min and 72 °C for 2 min; 38 cycles of 95 °C for 30 s, 58 °C for 1 min and 72 °C for 2 min; and 72 °C for 5 min; (b) PAS2, consisting of 95 °C for 5 min; two cycles of 95 °C for 30 s, 60 °C for 1 min and 72 °C for 2 min; 2 cycles of 95 °C for 30 s; 58 °C for 1 min and 72 °C for 2 min; two cycles of 95 °C for 30 s; 56 °C for 1 min and 72 °C for 2 min; 38 cycles of 95 °C for 30 s; 58 °C for 1 min and 72 °C for 2 min; and 72 °C for 5 min; and (c) PAS3, differing from PAS2 only in the extension step (68 °C for 3 min). PCR products were purified by treatment with ExonucleaseI (Fermentas) and Shrimp Alkaline Phosphatase (Fermentas), and Sanger-sequenced by Macrogen Europe (Amsterdam, The Netherlands). Mitochondrial DNA is usually overrepresented in Next Generation Sequencing (NGS) reads, allowing for easy retrieval of mitogenomes [45], as well as completion and curation of published mitochondrial sequences [23]. However, the correct assembly of insect mitogenomes from NGS data can be hampered by lack of adequate reference sequences, particularly in groups where gene rearrangements are common (e.g., Hymenoptera), and complete mitogenomes for some subfamilies are not available (e.g., Opiinae). Therefore, the mitogenome of *P. concolor* was sequenced using Sanger technology to obtain a reference sequence for subsequent mapping of the NGS reads from *P. humilis* and *P. lounsburyi.* The complete mitogenome sequence of *P. concolor* was recovered using a multi-step strategy starting with the PCR amplification of seed regions with primers designed on conserved regions of the mitochondrial genomes of *Diachasmimorpha longicaudata* (GenBank accession GU097655.1), *Spatius agrili* (GenBank accession NC_014278.1) and *Cotesia vestalis* (GenBank accession NC_014272.1). The seed regions were then iteratively extended using PCR primers specific for the newly obtained *P. concolor* sequences and primers based on the other Braconidae sequences, and the gaps between the seed regions were bridged using PCR primers specifically for *P. concolor*.

#### 2.2.2. Next Generation Sequencing

*Psyttalia humilis* and *P. lounsburyi* were sequenced using the Ion™ Torrent Proton™ platform (ThermoFisher Scientific, Waltham, MA, USA) available at the Central Analytical Facilities of Stellenbosch University, South Africa. Sequence libraries were prepared using the NEXTflex™ DNA Sequencing Kit for Ion Platforms (PerkinElmer, Waltham, MA, USA) according to the BI00 Scientific v15.12 protocol. Libraries were diluted to a target concentration of 60 pM. The diluted, barcoded libraries were combined in equimolar amounts for template preparation using the Ion PI™ Hi-Q™ Chef Kit (Thermo Fisher Scientific, Waltham, MA, USA). Twenty five microliters of diluted, pooled library was loaded onto the Ion Chef liquid handler (Thermo Fisher Scientific) for template preparation and enrichment using Ion PI™ Hi-Q™ Chef reagents, solutions and supplies according to the protocol, MAN0010967 REVB.0. Enriched ion sphere particles were loaded onto an Ion PI™ v3 chip. Massively parallel sequencing was performed on the Ion Torrent Proton system using sequencing solutions, reagents and supplies according to the protocol MAN0010967 REV B.0. Flow space calibration and basecaller analysis were performed using standard analysis parameters in the Torrent Suite version 5.10.0 software.

### 2.3. Mitogenome Assembly, Annotation and Analyses

The complete mitogenome of *P. concolor* was assembled using the CLCBio Main Workbench v6.9 (QIAGEN Bioinformatics), with manual curation. The NGS reads for *P. humilis* and *P. lounsburyi* were mapped and assembled to the complete mitogenome sequence of *P. concolor* using the mapper functionality available on Geneious Prime v2019.1 (https://www.geneious.com) with medium/low sensitivity option, and fine tuning up to five iterations. The consensus sequences were calculated using Geneious Prime. Open reading frames of protein-coding genes (PCGs) were identified using Geneious Prime, with the invertebrate mitochondrial genetic code. The position and secondary structure of transfer RNA genes (tRNAs) were predicted with ARWEN software [46] using the composite metazoan mitochondrial genetic code, and with MITOS WebServer (http://mitos.bioinf.uni-leipzig.de/index.py) using the invertebrate genetic code. Ribosomal RNA genes (rRNAs) were estimated by BLASTn search on NCBI (https://blast.ncbi.nlm.nih.gov). Overlapping regions and intergenic spacers were counted manually. Nucleotide composition and AT- and GC-skews were calculated using Geneious Prime, as AT-skew = (A − T)/(A + T) and GC-skew = (G − C)/(G + C). The sequences were deposited in GenBank under the accession numbers MW279212, MW279213 and MW279214.

### 2.4. Phylogenetic Analyses

The phylogenetic positioning of *P. humilis, P. concolor* and *P. lounsburyi* within Braconidae was reconstructed using 31 complete and partial mitogenomes of cyclostomes and non-cyclostomes, with two species of Ichneumonidae (*Diadegma semiclausum* and *Enicospilus* sp.) as outgroups (Table 1). In line with a previous study [37], analyses were restricted to all PCGs except ND2 due to incompleteness of several mitogenomes, and the first and second codon positions due to the temporal depth of the phylogeny. Sequences for each of the PCGs were aligned using the Translator-X server (translatorx.co.uk) [47], with alignment cleaning under less stringent selection and additional minor manual corrections. The 24 partitions corresponding to the first and second codon positions were separated using MEGA7 [48]. Subsequent analyses were performed using either the 24 partitions, or the 15-partition subset used by Li et al. (2016) [37], which was selected by excluding individual gene partitions with lower quality phylogenetic information. Three different partition-clustering schemes were tested for the datasets: (a) the partition scheme selected by PartitionFinder2 [49], run on the CIPRES Science Gateway V3.3 portal (www.phylo.org) [50] using a greedy algorithm (in line with Li et al. 2016); (b) a partition by codon position alone; (c) a partition by codon position and strand. In addition to facilitating comparisons with previous work, using PartitionFinder provides a “best-fit” (from a maximum likelihood perspective) partitioning scheme. However, it is generally advisable to test other partition-clustering schemes, particularly when phylogenetic analyses are conducted within a Bayesian framework. Partitioning by codon position alone or codon position + strand are commonly used in such comparisons as, on the one hand they have a biological basis, and on the other represent an intermediate between no partitioning and no partition clustering. Dated phylogenetic trees were obtained with a Bayesian method implemented in BEAST1.8.4 [51], with separate GTR + I + G (4 gamma categories) substitution models and lognormal relaxed clock models for each partition, but a single global tree model. The tree was left unconstrained except for monophyly requirements for both Braconidae and Ichneumonidae. A Yule process tree prior was used, and priors for divergence dates of Braconidae and Braconidae–Ichneumonidae were based on recently published data [52]. Priors for mutation rates were chosen based on previous results for insects [53], and values obtained with jModelTest2 [54]. Runs were performed for 30 million generations (main run: 15 partition data set, partition-clustering scheme selected by PartitionFinder) or 10 million generations (alternative runs, using the other partition-clustering schemes or/and the 24 partition data set), with a 10% generation burn-in and sampling every 1000 generations. Trees were summarized and annotated using TreeAnnotator v1.8.1 [51], and drawn using FigTree 1.4 (http://tree.bio.ed.ac.uk/software/figtree/).

## 3. Results and Discussion

### 3.1. Morphological and Molecular Species Identification

The adult specimens used for the recovery of the mitochondrial genomes of *P. concolor*, *P. humilis* and *P. lounsburyi* were identified morphologically by co-author V. Caleca. To confirm the morphological identification, intra- and interspecific genetic divergences using 650 bp of COI extracted for the new mitogenomes and all homologous sequences available on GenBank (*P. concolor,* n = 7; *P. humilis*, n = 10; *P. lounsburyi*, n = 32) were calculated. Intraspecific maximum p-distance was low in the three species (*P. concolor* = 0.32%; *P. humilis,* n = 0.71%; *P. lounsburyi*, n = 0.44%). Interspecific average p-distances were lowest for the pair *P. concolor*/*P. humilis* (4.76%; SE = 0.80), followed by *P. humilis/P. lounsburyi* (8.92%; SE = 1.16), and *P. concolor*/*P. lounsburyi* (9.66%; SE = 1.18). These results confirmed correct morphological identification of the specimens used for the recovery of the mitogenomes presented in the sections below.

### 3.2. Sequencing of the Psyttalia Mitogenomes

The complete mitogenome of *P. concolor* was recovered by Sanger sequencing of overlapping PCR-amplified fragments (see Material and Methods section for the amplification strategy), while the mitogenomes of *P. humilis* and *P. lounsburyi* were recovered using Ion Proton technology, resulting in 34.0 million reads (average read length of 177 bp) for *P. humilis*, and 11.6 million reads (average read length of 142 bp) for *P. lounsburyi*. Average sequence coverage was 304× for *P. humilis* and 285× for *P. lounsburyi*.

Hymenopteran mitogenomes are notoriously difficult to sequence due to very high A+T content, as well as frequent gene rearrangements. At the time of this study, sequences for all the mitochondrial genes had only been previously reported for two braconids (*S. agrili* and *C. vestalis*). In the present work, we obtained the sequences for all mitochondrial genes of *P. concolor*, *P. humilis* and *P. lounsburyi*. However, the AT-rich region was not successfully assembled for the last two species due to a combination of short NGS read length and extreme A+T content (90.9% in *P. concolor*). The AT-rich region had been previously annotated in only four braconids (*S. agrili*, *C. vestalis, D. longicaudata* and *Macrocentrus camphoraphilus*), and it is unclear whether it has been fully recovered in the last two species, as the reported mitogenomes lack the regions adjacent to ND2. Sequencing of the AT-rich region in Braconidae may be hampered not only by the typically high A+T content, but also by gene rearrangements and stable stem-and-loop structures commonly found in hymenopteran mitogenomes [55,56,57,58].

### 3.3. Organization and Composition of the Psyttalia Mitogenomes

#### 3.3.1. General Mitogenome Organization and Gene Content

**Figure 2:**
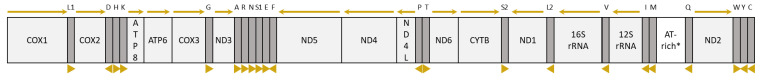
Organization of the mitochondrial genomes of *Psyttalia concolor, P. humilis* and *P. lounsburyi* (Hymenoptera: Braconidae). Transfer RNA genes are designated by the single-letter amino acid code. Arrows indicate the direction of gene transcription. AT-rich *-putative location of the control region.

The mitogenomes of the three *Psyttalia* had the typical metazoan gene content with 13 PCGs, two rRNAs, and 22 tRNAs, and gene arrangement was conserved among the three species (Table 2; Figure 2). Nine PCGs (*ND2, COI, COII, ATP8, ATP6, COIII, ND3, ND6, CTYB*) and 12 tRNAs (tRNA^Trp^, tRNA^Leu1^, tRNA^His^, tRNA^Lys^, tRNA^Gly^, tRNA^Ala^, tRNA^Arg^, tRNA^Asn^, tRNA^Ser1^, tRNA^Glu^, tRNA^Thr^, tRNA^Ser2^) were encoded on the majority strand (J-strand), and the remaining four PCGs (*ND5, ND4, ND4L, ND1*), 10 tRNAs (tRNA^Gln^, tRNA^Tyr^, tRNA^Cys^, tRNA^Asp^, tRNA^Phe^, tRNA^Pro^, tRNA^Leu2^, tRNA^Val^, tRNA^Ile^, tRNA^Met^) and the two rRNAs were encoded on the minority strand (N-strand). The tRNA^Gln^ region was poorly recovered in *P. humilis* and *P. lounsburyi* due to its location between the AT-rich region and ND2; therefore, this gene was only annotated in *P. concolor*.

The longest intergenic space averaged 164 bp across the three species and was located between COX2 and tRNA^Asp^. *Psyttalia* had few and short gene overlapping regions, with an average of 16 locations, and the longest overlap between ATP6 and ATP8 (22 bp).

#### 3.3.2. Nucleotide Composition and Strand Asymmetry

The three mitogenomes were highly biased towards A and T (average A+T content = 83.8), as typically is the case in insects (Table 3). The A+T content for PCGs on the N-strand (average = 83.8%) was higher than on the J-strand (average = 83.1%), with the highest in ATP8 (90.4%) in *P. humilis* and *P. lounsburyi,* and in ND6 in *P. concolor* (89.9%). The three *Psyttalia* had strand asymmetry with negative AT-skew (average = −0.06) and positive GC-skew (average = 0.19), similarly to the trend in other the 28 Braconidae (average AT-skew = −0.04, average CG-skew = 0.15) (Table 4). All genes in *Psyttalia* had negative AT-skews (PCG average = −0.07, tRNA average = −0.01 and rRNA average = −0.08), and positive GC-skews (PCG average = 0.20, tRNA average = 0.13 and rRNA average = 0.09). PCGs on the J-strand had negative AT-skew (−0.17) and positive GC-skew (average = 0.23), and PCGs on the N-strand had positive AT-skew (average = 0.83) and positive GC-skew (average = 0.14). Strand compositional bias (strand asymmetry) is frequent in insect mitogenomes, and is presumed to be the result of a prolonged single-stranded state of either the J-strand or the N-strand during transcription and replication, exposing one of the strands to a higher chance of DNA damage and repair. Exposed single-stranded DNA has a greater probability of deamination of C and A nucleotides, resulting in greater frequencies of C and A content on the complementary strand [36]. Consequently, positive AT-skew and negative GC-skew are usually observed on the J-strand. However, in some arthropods strand asymmetry is reversed, with negative AT-skews and positive GC-skews on the J-strand [58,59,60,61,62]. *Psyttalia* had strand asymmetry reversal on the J-strand, as had all other species in our dataset except *Proterops* sp. This feature may be explained by the inversion of the replication of origin in the AT-rich region in some insect lineages, as demonstrated previously [38].

### 3.4. tRNA Genes and Mitochondrial Gene Rearrangements in Braconidae

#### 3.4.1. tRNA Structure and Anticodons

The positions and structure of the tRNAs predicted by ARWEN and MITOS were identical. All tRNAs were predicted to fold into a cloverleaf structure except tRNA^Ser1^, for which the dihydrouridine (DHU) arm was reduced to a simple loop, a frequent occurrence in metazoans [63]. tRNA^Lys^ and tRNA^Ser2^ used the TTT and TCT anticodons instead of the regular CTT and GCT, respectively. In our dataset of mitogenomes, tRNA^Lys^ was annotated in all species and all used the TTT anticodon, except *Diadegma semiclausum* and *Enicospilus* sp. which used the regular CTT. tRNA^Ser2^ used the TCT anticodon in all species for which the gene was annotated (27/33). The usage of irregular anticodons in these two tRNAs could be associated with gene rearrangements [64].

#### 3.4.2. tRNA Rearrangements in Braconidae

**Figure 3:**
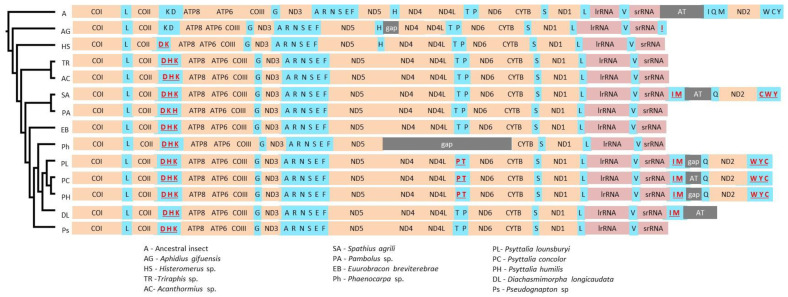
Gene arrangements in the mitochondrial genomes of 13 species of cyclostome wasps (Hymenoptera: Braconidae). Genes involved in rearrangements relative to the ancestral are shown in red, underlined. The species are ordered following the cyclostome tree presented in Figure 4.

Comparative mitogenomics have shown that gene rearrangements are infrequent in closely related taxa, and have a low likelihood of convergence owing to the large number of different possible combinations [65,66]. As such, mitochondrial gene rearrangements can be useful for resolving ancient evolutionary relationships [65,67]. The three *Psyttalia* shared the same gene arrangement but several differences relative to the other cyclostomes were present, all involving tRNAs (Figure 3). Twelve of the tRNA genes were organized into three clusters: D-H-K, A-R-N-S1-E-F and W-Y-C (genes in the N strand are underlined). In contrast, the ancestral insect mitogenome [68] is thought to have the A-R-N-S1-E-F, I-Q-M and W-C-Y organization, and the COX2 and ATP8 junction is COX2-K-D-ATP8. All *Psyttalia* had the derived state COX2-D-H-K-ATP8, with D inverted and having switched positions with K, and H inverted and translocated from its original position between ND5 and ND4 to its new location between D and K. Interestingly, H is found between COX2 and ATP8 in all cyclostomes except *Aphidius gifuensis* and *Histeromerus* sp., which occupy the most basal positions in the phylogeny of the clade (see below), suggesting that the translocation took place ~90 MYA. In congruence with the phylogeny, the basal *A. gifuensis* has preserved the ancestral K-D gene order, whereas *Histeromerus* sp. has the D-K arrangement. The different derived state COX2-D-K-H-ATP8, found in *Pambolus* sp., is therefore likely due to a subsequent exchange of positions between K and H.

In insects, the ancestral I-Q-M cluster is situated between the AT-rich region and ND2. All *Psyttalia* had the derived state I-M-AT-rich region-Q, with I and M inverted and translocated to their new position between 12S rRNA and the AT-rich region, a state which is shared with *S. agrili* and *C. vestalis.* Furthermore, the same topology might be present in *D. longicaudata* and *A. gifuensis* as they both have an inverted I (as well as M, in the case of *D. longicaudata)* adjacent to 12S rRNA, while the positions of the remaining elements are unknown due to incompleteness of the sequences. Interestingly, Ichneumonidae, the sister group to Braconidae, have the intermediate topology AT-rich region-I-M-Q. This suggests that the ancestral state in the common ancestor of Braconid–Ichneumonid is the state found in Ichneumonidae and raises the possibility that the srRNA-I-M gene arrangement is a synapomorphy of Braconidae.

In the insect ancestral mitogenome, the W-C-Y cluster is situated between ND2 and COX1. All *Psyttalia* had the derived state W-Y-C, in that C and Y swapped positions. This state is also found in species of Ichneumonidae, Pteromalidae and Eulophidae, while Orussidae and Vespidae have the ancestral state. If the W-Y-C state found in these different families had a common origin, it would conflict with a recent phylogenetic reconstruction of Hymenoptera [52]. However, a common origin seems unlikely as the ancestral state is conserved in the non-cyclostome *C. vestalis*, while a different derived state C-W-Y was found in *S. agrili*. As the sequence of this region has not been reported for the remaining 26 Braconidae included in our study, it is presently impossible to determine if the W-Y-C state found in *Psyttalia* is unique within the family. Remarkably, all *Psyttalia* have a unique rearrangement: the swapping of positions between P and T, which are situated between ND4L and ND6. This arrangement is not present in *D. longicaudata*; therefore, the feature could be specific to the genus *Psyttalia*.

### 3.5. Phylogenetic Position of Psyttalia within Braconidae

**Figure 4:**
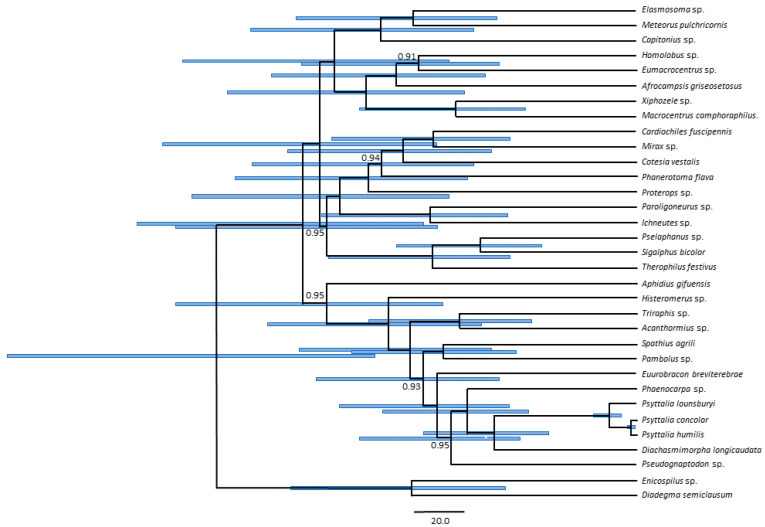
Bayesian reconstruction of the phylogenetic relationships among 31 species in the family Braconidae, with *Diadegma semiclausum* and *Enicosphilus* sp. (Ichneumonidae) as outgroups. Nodal support is given as Bayesian posterior probability (only values < 1 are shown). Scale bar represents 20 million years.

Phylogenetic analyses were performed primarily to determine the position of *Psyttalia* within Braconidae, and to obtain an estimate of the divergence times between the three species. The reconstruction was based on the set of 15 partitions from 11 PCGs used by Li et al. (2016) [37]. The *Psyttalia* species clustered with *D. longicaudata*, the only (partially) sequenced member of the subfamily Opiinae (Figure 4). The placement of *Psyttalia* was robust, as it was insensitive to the use of different datasets and partition-clustering schemes. The divergence between *Psyttalia* and *D. longicaudata* was estimated at ~55 MYA (95% HPD: 34–83 MYA). *Psyttalia lounsburyi* occupied the most basal position among the three *Psyttalia*, having diverged from the other two species ~11 MYA (95% HPD: 6–17 MYA). *Psyttalia concolor* and *P. humilis* were recovered as sister species, having diverged ~2 MYA (95% HPD: 1.1–3.6 MYA). These results support the taxonomic classification of *P. concolor* and *P. humilis* as distinct species, despite their high morphological similarity. These divergence times are also interesting from the perspective of gene rearrangements. Indeed, either a single (swapping of positions between the P and T tRNA genes) or two (if the swapping of positions between Y and C tRNAs is restricted to *Psyttalia*) rearrangements occurred between the divergence of *D. longicaudata* and the ancestor of *Psyttalia*, and none in the 11 MY after the divergence of the three species, suggesting that the timescale for gene rearrangements in Braconidae is in the order of tens of millions of years. Clarification of this matter will require a denser taxonomic coverage of mitogenomes of Braconidae.

Li et al. (2006) reported topological variations depending on the data matrices and analytical methods used in their analyses that included all available Braconidae mitogenomes. As such variations could potentially affect the phylogenetic position of *Psyttalia,* we conducted additional analyses using a data matrix containing 24 gene and codon partitions and/or different partition-clustering schemes. Some of the alternative analyses resulted in topological alterations, but none involved any of the Opiinae species (Figure S1). Surprisingly, the monophyly of the cyclostomes was sensitive to partition-clustering but not to the data matrix. *Aphidius gifuensis* was recovered as the most basal subfamily within cyclostomes when using the scheme selected by PartitionFinder, but it occupied the most basal position among all Braconidae in the trees obtained with the alternative partition-clustering schemes. Our analyses confirmed that some of the relationships among subfamilies of Braconidae are not recovered robustly, as previously reported [37]. The monophyly of cyclostomes was not robust, as the single available representative of Aphidiinae (*A. gifuensis*) was recovered as either basal within cyclostomes or basal within Braconidae, depending on the partition-clustering scheme used. This inconsistency and the smaller instabilities observed in the placement of other subfamilies should not be interpreted as evidence for the paraphyly of cyclostomes. Most probably, they reflect the difficulty in resolving phylogenies involving large time scales (almost 120 MYA) using mitochondrial sequences when only a small number of taxa is available. This limitation highlights the importance of increasing the mitogenomic coverage of Braconidae, particularly in subfamilies such as Aphidiinae, for which a robust phylogenetic position could not be determined at this point.

## Figures and Tables

**Table 1:** List of the partial and complete mitochondrial sequences used in phylogenetic reconstruction of the family Braconidae for the inference of the evolutionary relationships between *Psyttalia concolor, P. humilis* and *P. lounsburyi* and 28 other species of cyclostome and non-cyclostome wasps (Hymenoptera: Braconidae). *Diadegma semiclausum* and *Enicospilus* sp. (Hymenoptera: Ichneumonidea) were used as outgroups.

Species	Family	Subfamily	Lineage	GenBank	Reference	Size (bp)	Status
*Acanthormius* sp.	Braconidae	Lysiterminae	Cyclostome	KF385867.1	Li et al., 2016	13,051	Partial
*Afrocampsis griseosetosus* van Achterberg et Quicke, 1990	Braconidae	Acampsohelconinae	Non-cyclostome	KJ412474.1	Li et al., 2016	10,104	Partial
*Aphidius gifuensis* Ashmead, 1906	Braconidae	Aphidiinae	Cyclostome	GU097658.2	Wei et al., 2010	11,970	Partial
*Capitonius* sp.	Braconidae	Cenocoeliinae	Non-cyclostome	KF385869.1	Li et al., 2016	13,077	Partial
*Cardiochiles fuscipennis* Szépligeti, 1900	Braconidae	Cardiochilinae	Non-cyclostome	KF385870.1	Li et al., 2016	14,390	Partial
*Cotesia vestalis* (Haliday, 1834)	Braconidae	Microgastinae	Non-cyclostome	NC_014272.1	Wei et al., 2010	15,543	Complete
*Diachasmimorpha longicaudata* (Ashmead, 1905)	Braconidae	Opiinae	Cyclostome	GU097655.1	Wei et al., 2010	13,850	Partial
*Diadegma semiclausum* (Hellén, 1949)	Ichneumonidae	Campopleginae	-	NC_012708.1	Wei et al., 2009	18,728	Complete
*Elasmosoma* sp.	Braconidae	Euphorinae	Non-cyclostome	KJ412470.1	Li et al., 2016	12,326	Partial
*Enicospilus* sp.	Ichneumonidae	Ophioninae	-	FJ478177.1	Dowton et al., 2009	15,300	Partial
*Eumacrocentrus* sp.	Braconidae	Helconinae	Non-cyclostome	KF385872.1	Li et al., 2016	14,080	Partial
*Euurobracon breviterebrae* Watanabe, 1934	Braconidae	Braconinae	Cyclostome	KF385871.1	Li et al., 2016	12,957	Partial
*Histeromerus* sp.	Braconidae	Histerominae	Cyclostome	KF418765.1	Li et al., 2016	13,168	Partial
*Homolobus* sp.	Braconidae	Homolobinae	Non-cyclostome	KF385873.1	Li et al., 2016	13,927	Partial
*Ichneutes* sp.	Braconidae	Ichneutinae	Non-cyclostome	KF385874.1	Li et al., 2016	13,092	Partial
*Macrocentrus camphoraphilus* He et Chen, 2008	Braconidae	Macrocentrinae	Non-cyclostome	GU097656.1	Wei et al., 2010	15,801	Partial
*Meteorus pulchricornis* (Wesmael, 1835)	Braconidae	Euphorinae	Non-cyclostome	GU097657.1	Wei et al., 2010	10,186	Partial
*Mirax* sp.	Braconidae	Miracinae	Non-cyclostome	KJ412471.1	Li et al., 2016	13,664	Partial
*Pambolus* sp.	Braconidae	Pambolinae	Cyclostome	KF385875.1	Li et al., 2016	13,175	Partial
*Paroligoneurus* sp.	Braconidae	Ichneutinae	Non-cyclostome	KJ412472.1	Li et al., 2016	13,413	Partial
*Phaenocarpa* sp.	Braconidae	Alysiinae	Cyclostome	KJ412475.1	Li et al., 2016	9981	Partial
*Phanerotoma flava* Ashmead, 1906	Braconidae	Cheloninae	Non-cyclostome	GU097654.1	Wei et al., 2010	10,171	Partial
*Proterops* sp.	Braconidae	Ichneutinae	Non-cyclostome	KJ412477.1	Li et al., 2016	12,883	Partial
*Pselaphanus* sp.	Braconidae	Pselaphaninae	Non-cyclostome	KF385876.1	Li et al., 2016	13,204	Partial
*Pseudognaptodon* sp.	Braconidae	Gnamptodontinae	Cyclostome	KJ412473.1	Li et al., 2016	13,190	Partial
*Psyttalia concolor* (Szépligeti, 1910)	Braconidae	Opiinae	Cyclostome	MW279212	This study	15,308	Partial
*Psyttalia humilis* (Silvestri, 1913)	Braconidae	Opiinae	Cyclostome	MW279213	This study	15,311	Partial
*Psyttalia lounsburyi* (Silvestri, 1913)	Braconidae	Opiinae	Cyclostome	MW279214	This study	14,982	Partial
*Sigalphus bicolor* (Cresson, 1880)	Braconidae	Sigalphinae	Non-cyclostome	KF385878.1	Li et al., 2016	12,744	Partial
*Spathius agrili* Yang, 2005	Braconidae	Doryctinae	Cyclostome	NC_014278.1	Wei et al., 2010	15,425	Complete
*Therophilus festivus* Muesebeck, 1953	Braconidae	Agathidinae	Non-cyclostome	KF385868.1	Li et al., 2016	14,216	Partial
*Triraphis* sp.	Braconidae	Rogadinae	Cyclostome	KF385877.1	Li et al., 2016	13,162	Partial
*Xiphozele* sp.	Braconidae	Xiphozelinae	Non-cyclostome	KJ412476.1	Li et al., 2016	9160	Partial

**Table 2:** Main features of the mitochondrial genomes of *Psyttalia concolor, P. humilis* and *P. lounsburyi* (Hymenoptera: Braconidae). J-strand–majority strand, N-strand–minority strand; AC–anticodon; IGN–intergenic regions (+) and overlapping nucleotides (−). n. a.—not applicable.

		*Psyttalia concolor*						*Psyttalia humilis*						*Psyttalia lounsburyi*					
Gene/Region	Strand	Coordinates	Size	AC	Start	Stop	IGN	Coordinates	Size	AC	Start	Stop	IGN	Coordinates	Size	AC	Start	Stop	IGN
tRNA^Gln^	N	1–74	74	TTG	-	-	0	n.a.	n.a	n.a	-	-	n.a.	n.a.	n.a	n.a	-	-	n.a
ND2	J	78–1098	1021	-	ATA	T--	3	77–1097	1021	-	ATA	T--	n.a.	19–1039	1021	-	ATA	T--	n.a.
tRNA^Trp^	J	1099–1166	68	TCA	-	-	0	1097–1165	69	TCA	-	-	−1	1039–1107	69	TCA	-	-	−1
tRNA^Tyr^	N	1163–1228	66	GTA	-	-	−4	1162–1227	66	GTA	-	-	−4	1105–1168	64	GTA	-	-	−3
tRNA^Cys^	N	1228–1291	64	GCA	-	-	−1	1227–1290	64	GCA	-	-	−1	1170–1233	64	GCA	-	-	1
COX1	J	1292–2825	1534	-	ATG	T--	0	1291–2829	1539	-	ATG	TAA	0	1234–2772	1539	-	ATG	TAA	0
tRNA^Leu1^	J	2826–2893	68	TAA	-	-	0	2824–2891	68	TAA	-	-	−6	2767–2835	69	TAA	-	-	−6
COX2	J	2902–3558	657	-	ATA	TAA	8	2900–3558	657	-	ATA	TAA	8	2844–3500	657	-	ATA	TAA	8
tRNA^Asp^	N	3722–3791	70	GTC	-	-	163	3726–3795	70	GTA	-	-	168	3663–3736	74	GTC	-	-	162
tRNA^His^	J	3791–3858	68	GTG	-	-	−1	3795–3862	68	GTG	-	-	−1	3736–3804	69	CAC	-	-	−1
tRNA^Lys^	J	3858–3928	71	TTT	-	-	−1	3862–3932	71	TTT	-	-	−1	3804–3874	71	TTT	-	-	−1
ATP8	J	3929–4084	156	-	ATA	TAA	0	3933–4088	156	-	ATA	TAA	0	3875–4030	156	-	ATT	TAA	0
ATP6	J	4063–4752	690	-	ATT	TAA	−22	4067–4756	690	-	ATT	TAA	−22	4009–4698	690	-	ATT	TAA	−22
COX3	J	4762–5550	789	-	ATG	TAA	9	4766–5554	789	-	ATG	TAA	9	4702–5490	789	-	ATG	TAA	3
tRNA^Gly^	J	5551–5615	65	GGA	-	-	0	5555–5618	64	TCC	-	-	0	5491–5556	66	TCC	-	-	0
ND3	J	5630–6016	387	-	ATT	TAG	14	5633–6019	387	-	ATT	TAG	14	5571–5957	387	-	ATT	TAG	14
tRNA^Ala^	J	6015–6076	62	TGC	-	-	−2	6017–6079	63	TGC	-	-	−3	5955–6017	63	TGC	-	-	−3
tRNA^Arg^	J	6076–6142	67	TCG	-	-	-1	6079–6145	67	ACG	-	-	−1	6017–6083	67	ACG	-	-	−1
tRNA^Asn^	J	6136–6202	67	GTT	-	-	−7	6139–6205	67	AAC	-	-	−7	6077–6143	67	GTT	-	-	−7
tRNA^Ser1^	J	6200–6266	67	AGA	-	-	−3	6203–6269	67	AGA	-	-	−3	6141–6207	67	AGA	-	-	−3
tRNA^Glu^	J	6266–6330	65	TTC	-	-	−1	6269–6333	65	TTC	-	-	−1	6207–6271	65	GAA	-	-	−1
tRNA^Phe^	N	6329–6392	64	GAA	-	-	−2	6332–6396	65	GAA	-	-	−2	6720–6334	65	GAA	-	-	−2
ND5	N	6393–8052	1660	-	ATA	T--	0	6396–8055	1660	-	ATA	T--	−1	6334–7993	1660	-	ATA	T--	−1
ND4	N	8079–9401	1323	-	ATG	TAA	26	8082–9404	1323	-	ATG	TAA	26	8017–9342	1326	-	ATG	TAA	23
ND4L	N	9395–9691	297	-	ATT	TAA	−7	9398–9694	297	-	ATT	TAA	7	9336–9632	297	-	ATT	TAA	−7
tRNA^Pro^	N	9699–9765	67	TGG	-	-	7	9702–9768	67	TGG	-	-	7	9641–9708	68	TGG	-	-	8
tRNA^Thr^	J	9766–9829	64	TGT	-	-	0	9769–9832	64	TGT	-	-	0	9709–9772	64	TGT	-	-	0
ND6	J	9841–10,406	565	-	ATG	T--	11	9844–10408	565	-	ATG	T--	11	9784–10,350	567	-	ATG	TAA	11
CYTB	J	10,407–11,537	1131	-	ATG	TAA	1	10,410–11,540	1131	-	ATG	TAA	1	10,353–11,483	1131	-	ATG	TAA	2
tRNA^Ser2^	J	11,536–11,602	67	TGA	-	-	−2	11,539–11,605	67	TGA	-	-	−2	11,482–11,549	68	TGA	-	-	−2
ND1	N	11,601–12,560	960	-	ATT	TAA	−2	11,604–12,563	960	-	ATT	TAA	−2	11,548–12,507	960	-	ATT	TAA	−2
tRNA^Leu2^	N	12,561–12,626	66	TAG	-	-	0	12,568–12,629	62	TAG	-	-	4	12,508–12,574	67	TAG	-	-	0
16s RNA	N	12,627–13,914	1288	-	-	-	0	12,630–13,918	1289	-	-	-	0	12575–13,852	1278	-	-	-	0
tRNA^Val^	N	13,915–13,980	66	GTA	-	-	0	13,919–13,984	66	GTA	-	-	0	13,852–13,916	65	TAC	-	-	−1
12s RNA	N	13,981–14,727	747	-	-	-	0	13,985–14,729	745	-	-	-	0	13,918–14,671	754	-	-	-	1
tRNA^Ile^	N	14,728–14,791	64	GAT	-	-	0	14,730–14,793	64	GAT	-	-	0	14,672–14,735	64	ATC	-	-	0
tRNA^Met^	N	14,794–14,859	66	CAT	-	-	2	14,797–14,862	66	CAT	-	-	3	14,740–14,806	67	CAT	-	-	4
AT-rich region	-	148,69–15,308	449	-	-	-	0	n.a.	n.a.	-	-	-	0	n.a.	n.a.	-	-	-	-

**Table 3:** Nucleotide composition of the mitochondrial genomes of *Psyttalia concolor, P. humilis* and *P. lounsburyi* (Hymenoptera: Braconidae). PCGs–protein-coding genes. AT-skew = (A − T)/(A + T); GC-skew = (G − C)/(G + C).

		*Psyttalia concolor*								*Psyttalia humilis*								*Psyttalia lounsburyi*							
Region	Strand	A%	C%	G%	T%	A+T%	G+C%	AT-Skew	GC-Skew	A%	C%	G%	T%	A+T%	G+C%	AT-Skew	GC-Skew	A%	C%	G%	T%	A+T%	G+C%	AT-Skew	GC-Skew
COX1	J	30.2	9.8	15.8	44.0	74.4	25.6	−0.2	0.2	30.6	9.4	15.9	44.1	74.7	25.3	−0.2	0.3	30.7	10.2	15.1	43.9	74.7	25.3	−0.2	0.2
COX2	J	36.1	7.3	12.0	44.4	80.6	19.4	−0.1	0.2	35.3	7.0	12.6	45.1	80.4	19.6	−0.1	0.3	35.6	7.6	11.3	45.5	81.1	18.9	−0.1	0.2
ATP8	J	39.1	5.1	5.8	50.0	89.1	10.9	−0.1	0.1	37.8	4.5	5.1	52.6	90.4	9.6	−0.2	0.1	36.5	5.1	4.5	53.8	90.4	9.6	−0.2	−0.1
ATP6	J	33.5	7.8	8.3	50.4	83.9	16.1	−0.2	0.0	34.1	8.0	7.8	50.1	84.2	15.8	−0.2	0.0	32.2	8.6	8.7	50.6	82.8	17.2	−0.2	0.0
COX3	J	31.3	8.6	14.7	45.4	76.7	23.3	−0.2	0.3	30.7	8.7	15.6	45.0	75.7	24.3	−0.2	0.3	30.4	8.6	15.7	45.2	75.7	24.3	−0.2	0.3
ND2	J	36.2	3.2	7.6	52.9	89.1	10.9	−0.2	0.4	37.0	3.4	7.5	52.0	89.0	11.0	−0.2	0.4	37.1	3.4	7.4	52.0	89.1	10.9	−0.2	0.4
ND3	J	32.7	3.9	10.1	53.2	86.0	14.0	−0.2	0.4	33.1	3.9	10.1	53.0	86.0	14.0	−0.2	0.4	33.1	4.8	10.6	51.6	84.7	15.3	−0.2	0.4
ND5	N	45.4	6.2	9.5	38.9	84.3	15.7	0.1	0.2	44.8	6.5	9.7	39.1	83.9	16.1	0.1	0.2	44.7	6.3	10.0	39.0	83.7	16.3	0.1	0.2
ND4	N	44.8	7.0	10.1	38.1	82.9	17.1	0.1	0.2	44.9	6.9	9.8	38.4	83.3	16.7	0.1	0.2	44.4	7.3	9.9	38.4	82.8	17.2	0.1	0.2
ND4L	N	49.5	6.4	6.1	38.0	87.5	12.5	0.1	0.0	50.2	6.4	5.7	37.7	87.9	12.1	0.1	−0.1	49.2	6.7	6.4	37.7	86.9	13.1	0.1	0.0
ND6	J	38.7	3.7	6.4	51.2	89.9	10.1	−0.1	0.3	38.3	4.2	6.9	50.6	88.9	11.1	−0.1	0.2	39.0	4.2	6.9	49.9	88.9	11.1	−0.1	0.2
CytB	J	33.2	8.7	11.6	46.5	79.7	20.3	−0.2	0.1	33.2	8.8	12.2	45.8	79.0	21.0	−0.2	0.2	33.5	9.4	11.9	45.2	78.7	21.3	−0.1	0.1
ND1	N	45.0	9.1	9.9	36.0	81.0	19.0	0.1	0.0	44.3	9.1	10.4	36.3	80.5	19.5	0.1	0.1	44.3	9.3	10.2	36.3	80.5	19.5	0.1	0.0
12s rRNA	N	39.9	4.3	5.2	50.6	90.5	9.5	−0.1	0.1	39.5	4.3	5.6	50.3	90.1	9.9	−0.1	0.1	39.5	4.6	4.9	50.8	90.4	9.6	−0.1	0.0
16s rRNA	N	41.4	5.4	6.2	47.0	88.4	11.6	−0.1	0.1	41.2	5.4	6.1	47.2	88.4	11.6	−0.1	0.1	42.3	5.2	6.9	43.1	87.6	12.4	0.0	0.1
All PCGs	N+J	38.1	7.1	10.7	44.1	82.2	17.8	−0.1	0.2	38.0	7.1	10.8	44.0	82.0	18.0	−0.1	0.2	37.9	7.5	10.8	43.9	81.8	18.2	−0.1	0.2
All tRNAs		42.9	5.9	7.2	44.0	87.0	13.0	0.0	0.1	42.6	5.8	7.1	43.6	87.2	12.8	0.0	0.1	42.8	5.4	7.9	43.8	86.7	13.3	0.0	0.2
All rRNAs		40.8	5.0	5.8	48.3	89.1	10.9	−0.1	0.1	40.6	5.0	5.9	48.4	89.0	11.0	−0.1	0.1	41.3	5.0	6.2	46.0	88.7	11.3	−0.1	0.1
Complete mtDNA		39.4	6.5	9.5	44.6	84.0	16.0	−0.1	0.2	39.3	6.5	9.6	44.5	83.9	16.1	−0.1	0.2	39.0	6.8	9.8	44.4	83.4	16.6	−0.1	0.2

**Table 4:** AT-and GC-skews in the protein-coding genes (PCGs), tRNAs, rRNAs and the AT-rich region in the partial and complete mitochondrial genomes of 31 species in the family Braconidae. AT-skew = (A − T)/(A + T); GC-skew = (G − C)/(G + C).

	Total Sequence		PCGs		J-Strand PCGs		tRNAs		rRNAs	
Species	AT-Skew	GC-Skew	AT-Skew	GC-Skew	AT-Skew	GC-Skew	AT-Skew	GC-Skew	AT-Skew	GC-Skew
*Acanthormius* sp.	−0.11	0.19	−0.11	0.20	−0.23	0.25	−0.02	0.13	−0.14	0.04
*Afrocampsis griseosetosus*	0.44	0.31	−0.11	0.34	−0.18	0.34	−0.04	0.35	0.05	0.05
*Aphidius gifuensis*	−0.06	0.05	−0.06	0.07	−0.19	0.12	−0.02	0.00	−0.07	−0.10
*Capitonius* sp.	−0.07	0.19	−0.07	0.22	−0.20	0.25	−0.01	0.14	−0.09	−0.09
*Cardiochiles fuscipennis*	−0.07	0.18	−0.08	0.22	−0.20	0.25	−0.05	0.10	−0.06	0.01
*Cotesia vestalis*	−0.09	0.10	−0.11	0.12	−0.18	0.09	−0.03	0.11	−0.03	−0.11
*Diachasmimorpha longicaudata*	0.09	0.19	−0.10	0.21	−0.22	0.22	−0.03	0.17	−0.02	0.05
*Elasmosoma* sp.	−0.12	0.38	−0.14	0.40	−0.28	0.42	0.05	0.24	−0.07	0.16
*Eumacrocentrus* sp.	−0.01	0.05	−0.02	0.07	−0.13	0.10	0.01	0.07	0.03	−0.17
*Euurobracon breviterebrae*	−0.11	0.37	−0.13	0.39	−0.28	0.37	−0.03	0.32	−0.09	0.17
*Histeromerus* sp.	−0.06	0.16	−0.06	0.19	−0.18	0.19	−0.01	0.04	−0.09	−0.02
*Homolobus* sp.	−0.06	0.10	−0.06	0.10	−0.18	0.16	0.00	0.09	−0.06	−0.06
*Ichneutes* sp.	−0.06	0.21	−0.06	0.24	−0.08	0.25	−0.03	0.10	−0.09	−0.01
*Macrocentrus camphoraphilus*	−0.05	0.10	−0.06	0.13	−0.17	0.17	−0.01	0.02	−0.04	−0.11
*Meteorus pulchricornis*	−0.06	0.14	−0.06	0.16	−0.20	0.20	−0.02	0.15	-	-
*Mirax* sp.	−0.07	0.19	−0.07	0.02	−0.19	0.26	−0.04	0.22	−0.09	−0.06
*Pambolus* sp.	−0.09	0.16	−0.10	0.17	−0.22	0.20	−0.05	0.09	−0.08	0.00
*Paroligoneurus* sp.	−0.12	0.22	−0.12	0.25	−0.24	0.25	−0.04	0.08	−0.15	0.00
*Phaenocarpa* sp.	−0.09	0.11	−0.10	0.14	−0.19	0.17	0.00	0.17	−0.08	−0.08
*Phanerotoma flava*	−0.07	0.28	−0.07	0.29	−0.18	0.30	−0.01	0.15	-	-
*Proterops* sp.	0.06	−0.15	0.07	−0.14	−0.04	−0.10	0.03	0.00	0.06	−0.15
*Pselaphanus* sp.	−0.03	0.04	−0.03	0.08	−0.15	0.12	0.02	0.01	−0.05	−0.17
*Pseudognaptodon* sp.	−0.02	0.03	−0.02	0.04	−0.16	0.12	−0.03	0.11	−0.03	−0.18
*Psyttalia concolor*	−0.06	0.19	−0.07	0.20	−0.17	0.23	−0.01	0.10	−0.08	0.07
*Psyttalia humilis*	−0.06	0.19	−0.07	0.21	−0.17	0.24	−0.01	0.10	−0.09	0.08
*Psyttalia lounsburyi*	−0.06	0.18	−0.07	0.18	−0.17	0.20	−0.01	0.19	−0.05	0.11
*Therophilus festivus*	−0.03	0.02	−0.02	0.05	−0.15	0.09	0.01	0.01	−0.06	−0.13
*Triraphis* sp.	−0.12	0.19	−0.12	0.21	−0.25	0.19	−0.06	0.18	−0.17	−0.11
*Sigalphus bicolor*	−0.03	0.00	−0.02	0.02	−0.15	0.08	−0.02	0.07	−0.04	−0.18
*Spathius agrili*	−0.07	0.19	−0.07	0.20	−0.18	0.23	−0.04	0.13	−0.12	0.01
*Xiphozele* sp.	−0.01	−0.05	−0.02	−0.02	−0.09	0.01	0.02	−0.02	0.03	−0.27

## Supplementary Materials

The following are available online at https://www.mdpi.com/2075-4450/11/12/854/s1, Figure S1: Bayesian phylogenetic reconstruction among Braconidae obtained with different datasets and partition clustering schemes., Table S1: Primers and cycling protocols used for polymerase chain reaction (PCR) amplification and Sanger sequencing (Seq) of the complete mitochondrial genome of *Psyttalia concolor*.
