# Supplementary material for: New Mitochondrial Gene Rearrangement in Psyttalia concolor, P. humilis and P. lounsburyi (Hymenoptera: Braconidae), Three Parasitoid Species of Economic Interest
Source: Insects. 2020 Dec 2;11(12):854. doi: 10.3390/insects11120854 (PMC7761351; doi:10.3390/insects11120854)
Supplement: Supplementary file 1 [file insects-11-00854-s001.pdf]

Table S1. Primers and cycling protocols used for polymerase chain reaction (PCR) amplification and Sanger sequencing (Seq) of the complete mitochondrial genome of *Psyttalia concolor*. All segments were amplified and sequenced with primers designed specifically for this study, except S03* (universal DNA barcoding primers).

Segment	Cycling protocol	Primer	Primer sequence (5´- 3´)	Use-direction
S01	PAS2	TC16-023	TAG AAG TTT TAT TAG TGA TAA GTC	PCR-F, Seq-F
		TC14-172	TGT CTG ATT TAA GTA ATA AAC TG	PCR-R
S01	PAS2	TC15-107	ATA AAA TTA GGA ATA TTT CCC TTT C	PCR-F, Seq-F
		TC14-172	TGT CTG ATT TAA GTA ATA AAC TG	PCR-R
S02	PAS1	TC14-160	ATA TCA TTA GGT GGT TTA CCT CC	PCR-F, Seq-F
		TC11-107	GGT ATC CCT AAC TCT AAC CG	PCR-R, Seq-R
S03*	PAS1	LCO1490	GGT CAA CAA ATC ATA AAG ATA TTG G	PCR-F, Seq-F
		HC02198	TAA ACT TCA GGG TGA CCA AAA AAT CA	PCR-R, Seq-R
S04	PAS1	TC11-104	GGG ACC CTG TTT TAT ATC AAC	PCR-F, Seq-F
		TC14-016	CGA TTA TCA ACA TCT AAT AAA CG	PCR-R, Seq-R
		TC14-171	CTG CTA TAA TAG CAA ATA CAG C	Seq-R
S05	PAS1	TC14-108	AGG GCA TCA ATG ATA CTG AAG	PCR-F, Seq-F
		TC14-107	AAT GTA ATT AAT AAA TGA CCA GC	PCR-R, Seq-R
S06	PAS2	TC15-039	TAT ATT AGC TCA TTT AGT TCC TC	PCR-F
		TC16-018	AAC AAA ATG TCA ATA TCA TGA GG	PCR-R, Seq-R
S06*	PAS1	TC15-039	TAT ATT AGC TCA TTT AGT TCC TC	PCR-F, Seq-F
		TC15-074	AAA AAA CAG ACC CAT AAA TTG AAT C	PCR-R, Seq-R
S07	PAS2	TC15-098	GAG TAT TCT GAA TCA TAT TTT TC	PCR-F, Seq-F
		TC15-082	GTT TAA TAA TGA AAA TAT TAA GAT G	PCR-R, Seq-R
S08	PAS2	TC15-110	AAT TCC AAT TAA AAT AAG TAT GAA C	PCR-F, Seq-F
		TC15-101	TTA CAA AAA GAG CCC AAA TTC C	PCR-R, Seq-R
S09	PAS1	TC15-043	AGT TAC TAA TGT TGA GGA ATG AAC	PCR-F, Seq-F
		TC15-044	TGA TTA CCT AAA GCC CAT GTA GAA G	PCR-R, Seq-R
S10	PAS3	TC16-004	GAT TAA ACT AGA AAT TAA GCT TCC	PCR-F, Seq-F
		TC15-083	AAA TTA TTA ACC CAC CAA TAA TTA C	PCR-R, Seq-R
S10*	PAS2	TC15-076	AAC GGT ATA ATC CAT AAG ACC C	PCR-F, Seq-F
		TC15-083	AAA TTA TTA ACC CAC CAA TAA TTA C	PCR-R, Seq-R
S11	PAS2	TC15-084	CTG GCT TGA ATT CGA TCA ATT TG	PCR-F
		TC15-077	TAT GAA TTA AAC GTA TTA ATC ACC C	PCR-R
		TC16-015	ATAGATACAGGAATTGTTTTTAAC	Seq-F
S12	PAS3	TC15-102	TTT AGT TAA TTT ACC TAC TCC TG	PCR-F, Seq-F
		TC15-103	CTG CTT GAG ACC CTA ATC AAG	PCR-R, Seq-R
S13	PAS1	TC15-045	CTC CTG TTC ATA TTC AAC CAG AAT G	PCR-F, Seq-F
		TC15-046	TGC TCA ATC TAT TTC TTA TGA AG	PCR-R
S14	PAS2	TC14-162	ATT CAG ATT CTC CTT CTG ATA AAT C	PCR-F, Seq-F
		TC14-164	ATA AAT TAC CTT AGG GAT AAC AGC	PCR-R, Seq-R
S15	PAS2	TC15-085	GGC TTA CGC CGA TCT TAA CTC	PCR-F, Seq-F
		TC15-086	TTA ATA TAG GTA CAT ATC GCC CGT C	PCR-R, Seq-R
S16	PAS2	TC15-104	TTA GAA TTA TTC TGA TAC AAA AGG	PCR-F, Seq-F
		TC15-105	ATG AAG TGC CTG AAT AAA AGG	PCR-R, Seq-R
S17	PAS2	TC15-088	AAT AAT AGG GTA TCT AAT CCT AGT	PCR-F

		TC16-022	ATT TTA TCC CTA ATT TAA CTG CC	PCR-R, Seq-R
S17	PAS2	TC15-088	AAT AAT AGG GTA TCT AAT CCT AGT	PCR-F, Seq-F
		TC15-089	ATT TAA GCT TTT TGG TTC ATA CCC	PCR-R
S18	PAS2	TC15-106	AAT CCT TTT ATT CAG GCA CTT C	PCR-F, Seq-F
		TC15-109	CTC AAT TTA ACT CAA CTA ATA TAT C	PCR-R, Seq-R

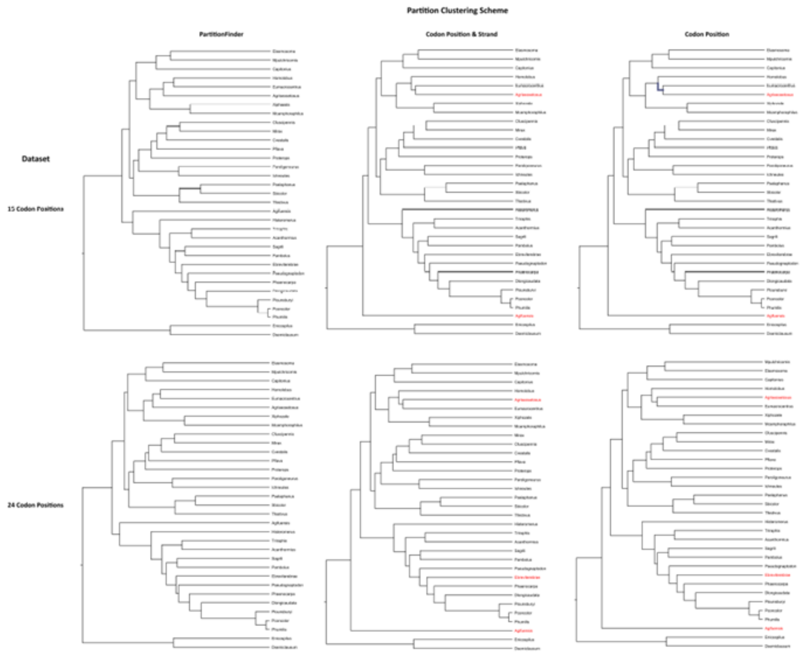

Figure S1. Bayesian phylogenetic reconstruction among Braconidae obtained with different datasets and partition clustering schemes. A to C: 15 codon positions dataset; D to F: 24 codon positions dataset; A and D: clustering with Partition Finder; B and E: clustering by codon position and strand location; C & F: clustering by codon position alone. Species with altered positions relative to the tree obtained with the main analysis (A) are indicated in red.
